# Resistance of Aerosolized Bacterial Viruses to Four Germicidal Products

**DOI:** 10.1371/journal.pone.0168815

**Published:** 2016-12-28

**Authors:** Nathalie Turgeon, Kevin Michel, Thi-Lan Ha, Enric Robine, Sylvain Moineau, Caroline Duchaine

**Affiliations:** 1 Centre de recherche de l’Institut universitaire de cardiologie et de pneumologie de Québec-Université Laval, Québec, Québec, Canada; 2 Département de biochimie, de microbiologie et de bio-informatique, Faculté des sciences et de génie, Université Laval, Québec, Québec, Canada; 3 Centre Scientifique et Technique du Bâtiment, Champs-sur-Marne, Marne la Vallée cedex, France; 4 Félix d’Hérelle Reference Center for Bacterial Viruses and GREB, Faculté de médecine dentaire, Université Laval, Québec, Québec, Canada; Universidade de Aveiro, PORTUGAL

## Abstract

Viral diseases can spread through a variety of routes including aerosols. Yet, limited data are available on the efficacy of aerosolized chemicals to reduce viral loads in the air. Bacteriophages (phages) are often used as surrogates for hazardous viruses in aerosol studies because they are inexpensive, easy to handle, and safe for laboratory workers. Moreover, several of these bacterial viruses display physical characteristics similar to pathogenic human and animal viruses, like morphological size, type of nucleic acids, capsid morphology, and the presence of an envelope. In this study, the efficacy of four chemicals was evaluated on four airborne phages at two different relative humidity levels. Non-tailed bacteriophages MS2 (single-stranded RNA), ϕ6 (double-stranded RNA, enveloped), PR772 (double-stranded DNA), and ϕX174 (single-stranded DNA) were first aerosolized in a 55L rotative environmental chamber at 19°C with 25% and 50% relative humidity. Then, hydrogen peroxide, Eugenol (phenylpropene used in commercial perfumes and flavorings), Mist^®^ (automobile disinfectant containing Triethylene glycol), and Pledge^®^ (multisurface disinfectant containing Isopropanol, n-Alkyl Dimethyl Benzyl Amonium Chlorides, and n-Alkyl Dimethyl Ethylbenzyl Ammonium Chloride) were nebulized with the phages using a separate nebulizer. Aerosols were maintained in suspension during 10 minutes, 1 hour, and 2 hours. Viral aerosols were sampled using an SKC BioSampler and samples were analyzed using qPCR and plaque assays. The resistance levels of the four phages varied depending on the relative humidity (RH) and germicidal products tested. Phage MS2 was the most stable airborne virus under the environmental conditions tested while phage PR772 was the least stable. Pledge^®^ and Eugenol reduced the infectivity of all airborne phages tested. At 25% RH, Pledge^®^ and Eugenol were more effective at reducing infectivity of RNA phages ϕ6 and MS2. At 50% RH, Pledge^®^ was the most effective agent against phage MS2. These findings illustrate that various airborne viruses should be tested to demonstrate the effectiveness of germicidal treatments. This research also provides a set of parameters for testing germicidal products in large-scale settings to reduce the risk of virus transmission.

## Introduction

Viral diseases can spread through a variety of routes such as direct contact with an infected person or indirect contact with fomites, exposure to large droplets, and inhalation of aerosolized droplets nuclei. The latter is of interest as they can stay in the air for an extended period of time and travel long distances. Airborne transmission of viruses has been demonstrated for some diseases, including measles [1] and smallpox [2]. However, the airborne transmission of other well-known viruses like *Influenza virus* and *Norovirus* is still under investigation [3, 4]. Indoor environments are particularly advantageous for viral transmission, including hospital settings where transmission of nosocomial diseases is a major concern [4–6]. Other public or occupational spaces such as day care centers and wastewater treatment plants are also suspected to represent occupational hazards with respect to viral diseases [7, 8].

When an environment is known or suspected to contain airborne pathogenic viruses, measures should be taken to minimize the risk of viral transmission and to reduce the viral load. Environmental factors such as temperature and relative humidity (RH) have a crucial impact on the infectivity of airborne viruses and can naturally help reduce viral concentrations in the air [9]. For example, infectivity of airborne *Influenza viruses* is minimal between 40% and 50% RH when compared to 20% and 70% RH [10]. Conversely, the infectivity of coronavirus is higher at 50% RH as compared to 20% and 80% RH [11]. Rhinovirus infectivity diminishes rapidly at 30% and 50% RH in comparison to 80% RH [12]. Therefore, the viral response to RH is virus-dependent.

Other environmental conditions or interventions should also be considered when trying to reduce airborne viral load. Experiments using ozone [13, 14] and UV light [15–19] have yielded promising results for inactivating airborne viruses. Studies conducted in controlled aerosol chambers demonstrated a significant reduction in virus infectivity using ozone or UV light for phages T7 [13, 19], MS2 [13–15, 18, 19], ϕ6 [13, 15, 19], ϕX174 [13, 15, 19], and PR772 [15] as well as for *Coronavirus* [18], *Adenovirus* [18], porcine reproductive and respiratory syndrome (PRRS) virus [16], and *Influenza virus* [17].

Gaseous disinfectant has been tested to inactivate viruses deposited on various surfaces at temperatures ranging from 25°C to 55°C as reviewed by Byrns and Fuller (2011). Others have tested the efficacy of numerous disinfectants against viruses at cold temperatures (-20°C to 4°C) [20]. Some investigators have looked at the potential of chemical solutions such as gaseous chlorine dioxide to kill aerosolized bacteria and fungi in indoor settings [21, 22]. However, chlorine dioxide is highly reactive (it’s difficult to transport, is an explosion hazard in gas form, can dissociate in to chlorine and oxygen during storage, etc.) and requires extreme safety precautions, which preclude its use for routine disinfection [23]. To our knowledge, there is very little (or no) information about the effectiveness of disinfectants against airborne viruses.

Bacterial viruses (bacteriophages or phages) are now widely used as models in aerosol studies [24]. They are relatively inexpensive to produce in large quantities and they do not require biosafety containment measures. Phages are highly diversified, and some of them possess structural similarities with human and animal viruses. Tailed phages with double-stranded DNA (dsDNA), like phages T4 and T7, were used in several previous aerosol studies [24]. However, since eukaryotic viruses lack tails, non-tailed phage models have been utilized more often in recent years. RNA phage MS2 (*Leviviridae* family) is now one of the most used models in viral aerosol studies [24]. Phage MS2 is highly resistant to aerosolization and sampling, and represents a good surrogate of *Newcastle disease virus* [25]. Phages ϕX174 (*Microviridae* family), ϕ6 (*Cystoviridae* family), and PR772 (*Tectiviridae* family) have also been requently used [13, 15, 25–29]. Previous studies have demonstrated that phages ϕ6 and PR772 displayed resistance to aerosolization and air sampling similar to *Influenza virus* [25]. Phage ϕ6 has been thought to be a good model for *Influenza virus* because it is an enveloped virus with a segmented RNA genome. The pattern of resistance to relative humidity exhibited by phage ϕ6 (resistant at low RH, very sensitive at middle RH, sensitive at high RH) is similar to what has been described for the *Influenza virus* [10, 15].

In a study by Verreault et al. (2015), a rotating environmental chamber was used to expose the above four phage models (MS2, ϕX174, ϕ6, and PR772) to 20%, 50% and 80% RH at 18°C and 37°C, for varying times up to 14 hours. Each phage reacted differently to environmental conditions. This suggests that this set of phages could be effectively used as viral simulants in bioaerosol studies. Here, we investigated the efficacy of four chemical or commercial products (hydrogen peroxide, Eugenol, MiST^®^, and Pledge^®^) to inactivate four airborne phage models using the same environmental chamber at 19°C, under 25% and 50% RH.

## Materials and Methods

### Bacteria and phages

Phages and bacterial host strains used in this study were obtained from the Felix d'Herelle Reference Center for Bacterial Viruses (www.phage.ulaval.ca) and are listed in Table 1. Tryptic soy broth (TSB) and tryptic soy agar (TSA) culture media were purchased from Difco Laboratory (Detroit, MI). Phages MS2 and ϕX174 were propagated on their bacterial host grown in TSB as described previously [26, 27]. Phage ϕ6 was grown on its host on TSA soft agar (0.75%) plates as reported elsewhere [27] while phage PR772 was amplified on TSB soft agarose (0.75%) plates [30]. Phage lysates were titrated on their respective bacterial hosts using plaque assays with TSA and TSB soft agar [31].

**Table 1:** Bacteria and phages used in this study.

Bacterial or viral strains	Characteristics and growth conditions	References
HER1036	*Escherichia coli*, TSB, 37°C, 200 rpm	[26]
HER1102	*Pseudomonas syringae* var. *phaseolicola*, TSB, 22°C, 100 rpm	[27]
HER1221	*E*. *coli*, TSB, 37°C, 200 rpm	[30]
HER1462	*E*. *coli*, TSB, 37°C, 200 rpm	[27]
HER36	Phage ϕX174, 25 nm diameter, nonenveloped, linear ssDNA genome, 5386 bases, bacterial host HER1036	[26]
HER102	Phage ϕ6, 85 nm, enveloped, segmented dsRNA, 13385 bp, bacterial host HER1102	[27]
HER221	Phage PR772, 80 nm, nonenveloped, linear dsDNA, 14492 bp, bacterial host HER1221	[30]
HER462	Phage MS2, 25 nm, nonenveloped, linear ssRNA, 3569 bases, bacterial host HER1462	[27]

ssDNA: single stranded DNA, ssRNA: single stranded RNA, dsDNA: double stranded DNA, dsRNA: double stranded RNA.

### Aerosolization experiments

The environmental chamber used in this study was composed of a cylindrical 55 L aluminum drum, mounted on double sealed ball bearings on both extremities. The internal sections of the bearings remain stationary during drum rotation. Probes and sampling ports were installed on the non-rotating part of the drum. The drum was installed in an insulated enclosure, in which the temperature could be controlled using thermoelectric assemblies. The environmental chamber is described in more detail by Verreault et al. (2014, 2015). The nebulization liquid was composed of 1 ml of each phage lysate (10^9^−10^10^ PFU/ml) and 100 μl of antifoam A (Sigma-Aldrich, Oakville, Ontario, Canada) to avoid foam in the nebulizer. Antifoam A has no effect on phages infectivity as demonstrated previously [15]. Nebulization liquid was completed to 50 ml with phage buffer (20 mM Tris-HCl, 100 mM NaCl, 10 mM MgSO4, pH 7.5). Phages were aerosolized using a 6-jet Collison nebulizer (BGI, Waltham, MA) driven by 20 psi compressed air for 10 minutes.

The four germicidal solutions tested were 3% hydrogen peroxide (Sigma-Aldrich), 10% Eugenol (phenylpropene used in commercial perfumes and flavorings, Sigma-Aldrich), MiST^®^ (automobile disinfectant containing 10% Triethylene glycol), and Pledge^®^ (multisurface disinfectant containing 1% Isopropanol, 0.0001% n-Alkyl Dimethyl Benzyl Amonium Chlorides, and 0.0001% n-Alkyl Dimethyl Ethylbenzyl Ammonium Chloride). The germicidal solutions were aerosolized using an Aeroneb Lab (Aerogen Inc, Galway, Ireland) filled with 5 ml of tested solution. After nebulization, the remaining volume of test chemical was measured in order to calculate the volume aerosolized. The concentrations of germicidal agents obtained in the aerosol chamber (C_T_, mg/L) are reported in Table 2.

**Table 2:** Antimicrobial agent concentration used in this study.

Chemicals	Concentration in nebulizer^c^ (mg/ml)	Volume aerosolized (ml)	Concentration in aerosol chamber^d^ (mg/L)
Hydrogen peroxyde	43.50	5.0	1.56
MiST (Triethylene glycol)^a^	125.50	1.0	0.90
Eugenol	10.67	0.3	0.02
Pledge^b^		0.3	
Isopropanol n-Alkyl Dimethyl Benzyl Ammonium Chloride	7.85 0.98		0.02 0.002

^a)^ MiST active ingredient is Triethylene glycol.

^b)^ Pledge two active ingredients: Isopropanol and n-Alkyl Dimethyl Benzyl Ammonium Chloride.

^c)^ Concentration of active ingredient in the nebulizer calculated from manufacturer specifications.

^d)^ Calculated according to Eq 1.

These concentrations were calculated according to Eq 1 [32] assuming perfect mixing and uniform concentration in the chamber, with the following parameters: chamber volume (V = 55 L), germicidal solution concentration (S, mg/ml), solution volume aerosolized (A, ml), air flow input (D = 12 L/min), air flow output equal to air flow input, nebulization time (T = 10 min), initial concentration in the chamber 0 mg/L.

$$C_{T}=\frac{SA}{TD}\left(1-e^{-\left(\frac{TD}{V}\right)}\right)$$ (1)

Diffusion dryers were used to remove excess humidity from the aerosol before it reached the chamber. Sixty-nine inches of diffusion dryer were used to get to 50% RH, and 108 inches were used to reach 25% RH. Chamber rotation was set at 1 rpm to keep aerosols in suspension. Aerosols remained suspended in the rotating chamber for 10 minutes, 1 hour and 2 hours. For each experiment, the temperature was set to 19°C in order to maintain a temperature range between 18°C and 22°C. Relative humidity and temperature were recorded using a probe (model RH-USB, Omega) installed inside the rotating chamber. Particle size distribution and concentration were measured with an Aerodynamic Particle Sizer (APS) (model 3321, TSI Inc., Shoreview, MN) equipped with a 1/100 diluter (model 3302A, TSI Inc.) immediately after aerosolization, after 10 minutes stabilization, and before sampling.

Aerosols were sampled using SKC BioSampler (SKC Inc., Eighty Pour, PA) filled with 20 ml of phage buffer, for 20 min at 12.5 l/min (determined by the critical orifice of the instrument). The air was driven into the SKC BioSampler using an SKC pump (model 228–9605). Air samples and nebulizer solutions were analyzed using qPCR and plaque assays. Samples were stored at 4°C, for no more than 3 hours, until they were analyzed using plaque assays. Aliquots were stored at –20°C for RNA extraction and qPCR analysis (3–6 days). During each aerosolization experiment, the chamber was filled and allowed to stabilize for 10 minutes. Aerosols were then sampled immediately (time point 0 h exposure), or kept in the chamber for 1 or 2 hours. During air sampling, aerosol concentrations in the chamber followed an exponential decay. Fifty percent of the aerosols were collected during the first three minutes of air sampling with the SKC BioSampler as calculated with Eq 2 [32]. The initial concentration was considered to be 100% (C_i_), the air input and output were 12.5 L/min, and the aerosol input was clean HEPA filtered air. During twenty minutes of air sampling with the SKC BioSampler 99% of the aerosols were collected from the chamber. Therefore, the chamber had to be filled for every time point tested, and thus, each set of test conditions was considered a separate experiment. All tested conditions and time points were repeated twice. Phages were also aerosolized without germicidal agents as control experiments. Nebulizer content was analyzed using a plaque assay before and after aerosolization to measure phage infectivity over the duration of the experiments.

$$C_{T}=C_{i}(e^{-(\frac{TD}{V})})$$ (2)

### Blank experiments and chamber cleaning

The chamber was purged for 30 minutes with 20 L/min clean medical grade air before each aerosolization experiment. No particles were detected using the APS after purging. All tubings were disconnected from the chamber and cleaned daily to avoid phage carryover between experiments. Blank experiments were conducted by aerosolizing phage buffer without any phage to make sure that the purge procedure and cleaning process were efficient. The samples from these blank aerosolizations were processed using the identical procedures as for the other samples.

### RNA extraction and qPCR

The genomic RNA of phages ϕ6 and MS2 was extracted and reversed transcribed into cDNA as previously described [27]. Briefly, phage RNA was extracted using QIAamp viral RNA minikit. RNA carier was omitted from the Qiagen AVL buffer and the RNA was eluted with 2 volumes of 40 μl of TE buffer (10 mM Tris, 0.1 mM EDTA). RNA was stored at –80°C or processed immediately for cDNA synthesis. RNA was heated (100°C, 5 minutes) before performing cDNA synthesis using iScript cDNA Synthesis kit (Bio-Rad Laboratories) following the manufacturer instructions using 10 μl of asamples. Primers and probes used for qPCR reactions are listed in Table 3 and were provided by Integrated DNA Technologies (IDT). Probes were labeled with FAM dye in 5’ and with a combination of Zen and Iowa black FQ quencher in 3’. Quantitative PCR assays for phages ϕ6 and MS2 [27] and phages PR772 [25] and ϕX174 [26] have been developed in previous studies. Complete protocols for qPCR detection for these four phages can be found in the following reference [15]. Briefly, each 20 μl reaction contained the following: 10 μl of 2X iQ supermix for probe (Bio-Rad Laboratories), 2 μl of cDNA (MS2 and ϕ6) or 5 μl of sample (PR772 and ϕX174), and 1 μM of forward and reverse primers. The reaction mix also contained the following probe concentrations: 150 nM, 300 nM, 200 nM and 200 nM for MS2, ϕ6, ϕX174, and PR772, respectively. Reactions were performed using a BioRad CFX 96 or BioRad CFX384. The PCR program was as follows: 95°C for 5 minutes followed by 39 cycles of 95°C for 10 seconds, 60°C for 30 seconds, and fluorescence measurements.

**Table 3:** Primers and probes used in this study.

Primers	Sequences (5’-3’)	Target (genome position)	Ref
ϕX174for	ACAAAGTTTGGATTGCTACTGACC	ϕX174 (508–531)	[26]
ϕX174rev	CGGCAGCAATAAACTCAACAGG	ϕX174 (630–609)	[26]
ϕX174probe	FAM/CTCTCGTGC/ZEN/TCGTCGCTGCGTTGA/IABlkFQ	ϕX174 (533–556)	[26]
ϕ6Tfor	TGGCGGCGGTCAAGAGC	ϕ6 (S, 430–446)	[27]
ϕ6Trev	GGATGATTCTCCAGAAGCTGCTG	ϕ6 (S, 530–506)	[27]
ϕ6Tprobe	FAM/CGGTCGTCG/ZEN/CAGGTCTGACACTCGC/IABlkFQ	ϕ6 (S, 450–474)	[27]
PR772for	CCTGAATCCGCCTATTATGTTGC	PR772 (4538–4560)	[25]
PR772rev	TTTTAACGCATCGCCAATTTCAC	PR772 (4663–4641)	[25]
PR772probe	FAM/CGCATACCA/ZEN/GCCAGCACCATTACGCA/IABlkFQ	PR772 (4639–4614)	[25]
MS2 1for	GTCCATACCTTAGATGCGTTAGC	MS2 (1261–1284)	[27]
MS2 1rev	CCGTTAGCGAAGTTGCTTGG	MS2 (1420–1401)	[27]
MS2 1probe	FAM/ACGTCGCCA/ZEN/GTTCCGCCATTGTCG/IABlkFQ	MS2 (1391–1367)	[27]

### Data calculation

Plaque assays were used to measure infectious phages in all samples. In previous experiments, it has been demonstrated that viruses genomes are stable under air sampling conditions used in this study [15, 25, 33], therefor, qPCR was used to measure total phage genomes in all samples. The ratio of infectious phages in air samples was calculated by dividing the number of infectious phages as determined by plaque assays by the number of phage genomes estimated by qPCR. Because infectious ratio is affected by RNA extraction, cDNA synthesis and qPCR efficiency [27] as well as by aggregates in plaque assays, the value may be below or above 1. These assays bias are phage-specific and therefore, infectious ratios should not be used to compare phages. However, infectious ratios can be used to compare the effect of environmental conditions (relative humidity and exposure time) on each phage since the limitations are likely the same for all samples of the same phage.

The effect of germicidal agents was expressed as a relative ratio of the infectious phages ratio aerosolized with chemicals divided by infectious phages ratio aerosolized without chemicals as displayed in Eq 3.

RelativeInfectiousRatio=(PFU/mlgenomes/ml)WithVirucide(PFU/mlgenomes/ml)WithoutVirucide (3)

### Statistical analysis

Data were expressed using mean ± sd or median (interquartile range) to summarize assay characteristics. Data were analyzed using a two-way ANOVA with an interaction effect. ANOVAs were fitted to compare heterogeneous variances among levels of humidity or “commercial products” with three time periods and were tested if the models could be reduced to ANOVAs with the same variance across factor levels. The univariate normality assumption was verified using the Shapiro-Wilk tests on the error distribution from the statistical model after a Cholesky factorization. The Brown and Forsythe's variation of Levene's test was used to verify the homogeneity of variances. When appropriate, some variables were log-transformed to fulfill the model assumptions. Reported p-values were based on these transformations. When these assumptions were not fulfilled, an alternative procedure called rank transformation was used. In a rank transformation the observations are replaced by their rank and an ordinary F-test from ANOVA is applied. This analysis does not depend on the assumptions required by the Brown and Forsythe’s variation of Levene’s test. This technique resulted into good statistical properties when compared to standard test. When results from raw or log-transformed data were compared with ranks and yielded similar results, data from the standard analyses were retained. When results differed, the rank transformation data was preferred. Posteriori comparisons were performed using Tukey’s comparison test. Results were considered significant when p-values ≤ 0.05. Data were analyzed using the statistical package program SAS v9.4 (SAS Institute Inc.).

## Results

### Aerosol characteristics

Particle concentration in the aerosol chamber ranged between 4.8 x 10^4^ and 1.2 x 10^5^ particles per cubic centimeter. The median mass aerodynamic diameter (MMAD) was between 0.9 and 1.1 μm immediately after aerosolization. Particle concentration and MMAD inside the chamber were in accordance with previous data [34]. Recorded temperature and relative humidity varied slightly within and between experiments. For all of the experiments, the temperature fluctuated between 18°C and 21.3°C. The relative humidity levels ranged from 24.6% to 26.5% for experiments set at 25% RH, while RH ranged from 48.2% to 52.4% when set at 50%.

### Effect of relative humidity on the infectivity of airborne phages

**Fig 1:**
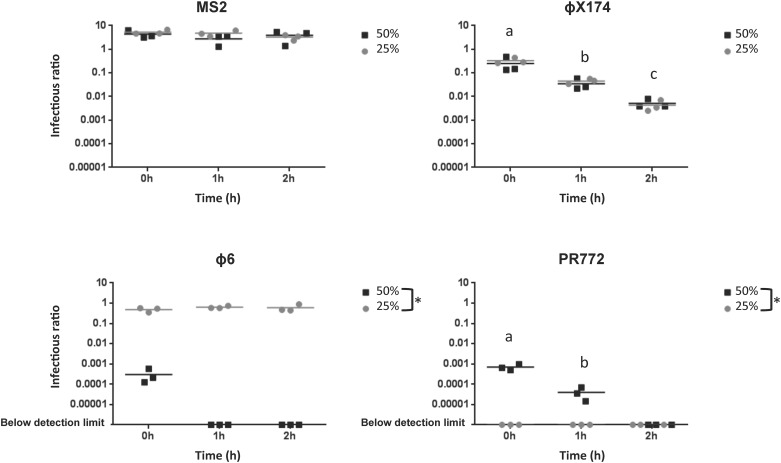
Effect of relative humidity and exposure time on four aerosolized phage models. Experiments were conducted at 19°C with 25% (black circles) and 50% (black squares) relative humidity. a, b, c indicate significant effect of exposure time for phages ϕX174 and PR772. The asterisk (*) indicates a significant difference between 25% and 50% relative humidity for phages ϕ6 and PR772.

Control experiments were conducted to evaluate the effect of 10 minutes, 1 hour, and 2 hours of exposure to 25% and 50% RH on the infectivity of aerosolized phages (Fig 1). No significant effect of RH or exposure time was observed for phage MS2, confirming its stability in an airborne state. However, there was a significant effect of exposure time on phage ϕX174 at both 25% and 50% RH (p<0.0001), suggesting that this phage cannot persist for a long period of time in airborne state. Finally, we observed a significant difference between the exposure to 25% and 50% RH for phages ϕ6 and PR772 (p<0.0001). Phage ϕ6 was highly stable when exposed to 25% RH. However, it was very unstable at 50% RH, suggesting a preference for a less humid environment. Conversely, phage PR772 was highly unstable at 25% RH as plaque assay results were below the detection limit for all exposure times. PR772 was also highly unstable at 50% RH after 1 hour and 2 hour exposure times, suggesting that this phage is not well adapted to these environmental conditions. Due to this instability experiments using Phage PR772 were discontinued.

### Effect of germicidal agents on relative infectivity ratios

**Fig 2:**
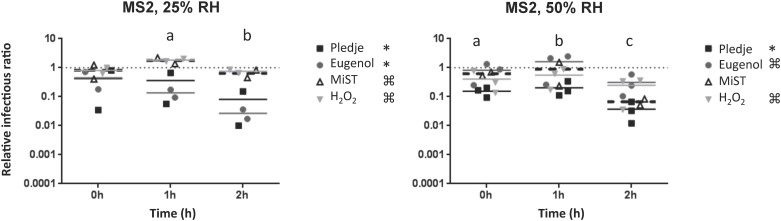
Effect of Pledge^®^ (black squares), Eugenol (gray circles), MiST^®^ (white triangles), and H2O2 (gray inverted triangles) on the infectivity of airborne phage MS2. Experiments were conducted at 19°C with 25% and 50% RH. The dotted line indicates the reference value without a chemical agent for the same RH and exposure time. a and b indicate a significant effect of exposure time for all chemical agents on MS2 relative infectivity ratios. The asterisk (*) and ⌘ indicate significant differences between the effects of Pledge^®^ and MiST^®^, Pledge^®^ and H_2_O_2_, Eugenol and MiST^®^, Eugenol and H_2_O_2_ on MS2 infectivity ratios at 25% RH, and significant differences between the effects of Pledge^®^ and Eugenol, and Pledge^®^ and H_2_O_2_ on MS2 infectivity ratios at 50% RH.

**Fig 3:**
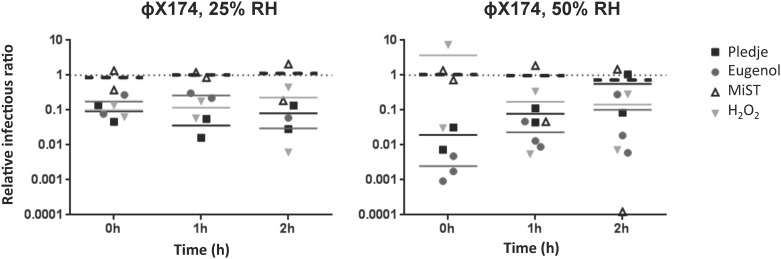
Effect of Pledge^®^ (black squares), Eugenol (gray circles), MiST^®^ (white triangles), and H2O2 (gray inverted triangles) on the infectivity of airborne phage ϕX174. Experiments were conducted at 19°C with 25% and 50% RH. The dotted line indicates the reference value without a chemical agent for the same RH and exposure time. No significant effect of exposure time or of chemical agent was observed.

**Fig 4:**
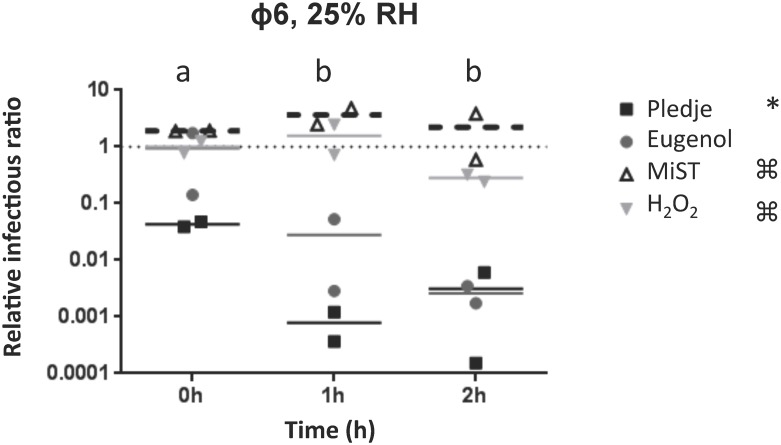
Effect of Pledge^®^ (black squares), Eugenol (gray circles), MiST^®^ (white triangles), and H2O2 (gray inverted triangles) on the infectivity of airborne phage ϕ6. Experiments were conducted at 19°C with 25% and 50% RH. Results obtained with 50% RH were below detection limit. The dotted line indicates the reference value without a chemical agent for the same RH and exposure time. a and b indicate a significant effect of exposure time for Pledge^®^ on phage ϕ6 infectivity. Asterisk (*) and ⌘ indicate significant differences between the effect of Pledge^®^ and MiST^®^ and between Pledge^®^ and H_2_O_2_.

Once airborne, the effects of germicidal products, RH, and exposure time on phage infectivity are likely cumulative. The levels of infectious phage particles were determined after the exposure of four airborne phages to four germicidal products at 25% and 50% RH and compared to the reference values obtained without germicidal product at the same RH level and exposure times. As indicated in the Materials and Methods section, the data are presented as relative infectivity ratios, which allows for the analysis of the germicidal product effect alone without interference of the RH. The reference values obtained without germicidal product (relative infectivity ratio = 1) are represented by the dotted lines in Figs 2–4.

All germicidal products had a significant effect on phage MS2 relative infectivity ratios at 25% RH (p<0.003) and 50% RH (p<0.005) (Fig 2). The duration of chemical exposure also had a significant impact on phage MS2, particularly at 50% RH (p<0.003). Although a reduction of MS2 infectious particles was noticed between 1 and 2 hours of exposure with all germicidal products at 25% RH, the effect of exposure time was not significant under these conditions (p = 0.06). At 25% RH, 1 hour and 2 hour exposure times using Pledge^®^ and Eugenol were 5–10 times more efficient than MiST^®^ and H_2_O_2_ at reducing the relative infectious ratios of phage MS2 (p<0.03). At 50% RH, Pledge^®^ reduced the relative infectious ratios of phage MS2 5 to 10 times more than MiST^®^ and Eugenol (p<0.05). Overall, a maximum reduction of less than two orders of magnitude was noted under all conditions tested with phage MS2.

Phage ϕX174 infectious particles were reduced one order of magnitude when exposed to Pledge^®^ and Eugenol at 25% RH (Fig 3). At 50% RH, 10 minutes of exposure to Pledge^®^ or Eugenol reduced phage ϕX174 infectious particles by 50 and 500 fold compared to the reference values without germicidal product. However, after 1 hour and 2 hours of exposure at 50% RH, the reduction of phage ϕX174 was less pronounced (Fig 3).

Phage ϕ6 infectious particles were reduced 10 to 1000 fold at 25% RH when exposed to Pledge^®^ (Fig 4)(p<0.006). Pledge^®^ was significantly more effective than MiST^®^ and H_2_O_2_ in reducing phage ϕ6 relative infectious ratios (p<0.001). All plaque assay results were below the detection limit with phage ϕ6 at 50% RH.

## Discussion

### Effect of relative humidity

Aerosolization and air sampling puts significant stress on microorganisms. Environmental conditions and time spent airborne are also critical factors affecting the survival of microbes and prolonged infectivity of viruses. Viruses like *Influenza*, *Murine Norovirus*, *Adenovirus*, *Coronavirus*, as well as several phages are known to lose infectivity over time once airborne [4, 15, 18, 25]. Therefore, these factors must be also considered when measuring the efficacy of germicidal agents on airborne microorganisms and viruses.

In a previous study [15] we demonstrated that temperature (18°C, 37°C), RH (20%, 50%, 80%), and time spent airborne (0h, 1h, 6h, 14h) affected the infectivity of airborne phages MS2, PR772, ϕX174, and ϕ6 differently. These earlier experiments were conducted using the same aerosol chamber, nebulizer, and air sampler used in the current study. However, few parameters were changed. The addition of a second nebulizer for germicidal agents aerosolization forced setup modification compared to the precedent study. Aerosolized phages were passed through diffusion dryers to remove humidity before being mixed with aerosolized agents in a second set of diffusion dryers. The lowest RH that could be reached in experiments using H_2_O_2_ and MiST^®^, and in which 5 ml of germicidal product were aerosolized, was 25%. Therefore, the control experiments without agents were conducted at 25% RH. These controls without agents are very important because they set the reference value used to evaluate the germicidal effect of the agents as explained in Eq 3.

Although the environmental conditions tested were variable, our results were consistent with previous studies, indicating data reproducibility. For example, it was previously observed that phage MS2 is highly stable in aerosol state [15, 18, 25, 27]. Our results also confirm that aerosolized phage PR772 is more infectious at middle and high RH levels (50% and 80%) compared to low RH levels (20% or 25%, [15] whereas phage ϕ6 was more resistant at low RH (20% or 25%) compared to medium (50%) and high RH [15]. Previously, phage ϕX174 was found to be more resistant to 80% RH compared to 20% and 50% [15]. Although 80% RH exposure was not tested in here, we did not observe significant differences between infectivity at 25% and 50% RH.

The above data clearly indicate that resistance to environmental conditions is virus dependent. Yet, *Adenovirus* and *Rhinovirus* were shown to be infectious for longer periods of time when exposed to high RH levels, similar to phages PR772 and ϕX174 [12, 35]. However, it is well documented that *Influenza viruses* are more stable at low RH levels, as observed for phage ϕ6 [36, 37]. Phage MS2 was the most stable airborne virus under the conditions tested here (Fig 1). Overall, the above data support the need for testing various phages with different properties in studies that examine aerosols.

### Effect of germicidal products

Pledge^®^ (containing Isopropanol, n-Alkyl Dimethyl Benzyl Amonium Chlorides, and n-Alkyl Dimethyl Ethylbenzyl Ammonium Chloride) and Eugenol reduced the relative infectious ratios of all airborne phages tested. However, the effect was only statistically significant for the two RNA phages, MS2 and ϕ6 (Figs 2 and 3). Surprisingly, the relative infectious ratios of phage ϕ6 were above one when aerosolized with MiST^®^ (containing Triethylene glycol), which may suggest a protective effect on phage infectivity (Fig 3). Experiments using phage ϕX174, showed that the relative infectious ratios were below one with Pledge^®^, Eugenol, and H_2_O_2_ at 25% RH and with Pledge^®^ and Eugenol at 50% RH (Fig 4). These data indicate that the chemicals reduced the infectivity of airborne phage ϕX174 compared to the reference values without germicidal product. The time spent airborne also has a strong effect on phage ϕX174 infectivity (Fig 1). For 1 hour and 2 hours of exposure at 50% RH, the impact of germicidal agents on the reduction of phage ϕX174 relative infectious ratios was less pronounced due to the effect of increased exposure time (Fig 4). The effects of germicidal agents on the four phages are summarized in Table 4. Others have also observed a virus-dependent response to sanitizing conditions. *Coronavirus* was shown to be 7–10 times more vulnerable to UV than *Adenovirus* and phage MS2 [18]. Phages T7 and MS2 were more resistant to ozone than ϕ6 and ϕX174 [13], while T7 was more resistant to UV than MS2, ϕ6, and ϕX174 [19].

**Table 4:** Behavior of the four phages under tested conditions.

Phage	25% HR					50% RH				
	No agent	Pledge	Eugenol	MiST	H_2_O_2_	No agent	Pledge	Eugenol	MiST	H_2_O_2_
MS2	+	--	--	-	-	+	--	-	-	-
ϕX174	-	+	+	+	+	-	+	+	+	+
ϕ6	+	-	+	+	+	-	ND	ND	ND	ND
PR772	ND	NA	NA	NA	NA	-	NA	NA	NA	NA

+: no significant effect (resistant), -: significant effect (sensitive), --: more effect compared to other agents at same RH, ND: not detected, NA: not available

The duration of nebulization using the Aeroneb Lab depends on the contents of the aerosolized solution. For example, ethanol- and isopropanol-based solutions (>30%) are slowly aerosolized compared to water-based solutions (more than 7 times longer to aerosolize the same volume). The Aeroneb Lab is widely used in drug delivery assays because it is non-destructive, can be used between 5 to 30 liters of air per minutes without influencing the liquid volume nebulized, and can aerosolise >0.2 ml of water-based solution per minute [38]. Since the composition of each of the germicidal agents used was different, the volume aerosolized and the concentration reached in the chamber for each agent was also different (Table 2). In our experimental setup it was necessary to fit the germicidal nebulization time within the phage’s nebulization times. The nebulization time was set to 10 min in order to reach the same phage concentration in the chamber for all the assays. Because the infectivity of phages PR772 and ϕX174 is strongly affected by their time spent airborne, it was essential to standardize nebulization times, germicidal exposure, and sampling times. Therefore, it was not possible to extend the aerosolization time to increase the concentration of the germicidal agent in the chamber. Despite lower concentrations of germicides in the chamber compared to MiST^®^ and H_2_O_2_, Pledge^®^ and Eugenol led to the highest reduction (up to 3 logs) of airborne viruses.

Previous studies have demonstrated that 10–20 seconds of exposure to UV light or ozone reduces 90% of the remaining infectious viruses in an aerosol chamber [13, 15]. Since the doses tested are harmful to humans, these methods could be used in ventilation ducts or ceilings but not in occupied spaces. In the current study, two hours of exposure to Pledge^®^ or Eugenol were necessary for a 1-log reduction of infectious phage MS2. For phages ϕ6 and ϕX174, this 1-log reduction was achieved after only 10 minutes (time 0) of exposure to Pledge^®^ and Eugenol.

The average limit of exposure to H_2_O_2_ over an eight hour work shift (TWA) recommended by the ACGIH is 1 ppm (http://www.cdc.gov/niosh/idlh/772841.html). The concentration of H_2_O_2_ used in this study (1209 ppm) exceeds the ACGIH TWA guidelines by more than 1200 ppm and produced poor results for reducing phage infectivity. Trietylene glycol (contained in MiST^®^) is one of the products used in fog machines to produce a vapour similar to fog or smoke. Exposure limits have not been established for Trietylene glycol. However, the Threshold limit value (TLV) recommended by ACGIH for Ethylene glycol (another component used in fog machines) is 100 mg/m^3^ (https://www.osha.gov/dts/chemicalsampling/data/CH_240404.html). The concentration used in this study (900 mg/m^3^) exceeds this recommendation by nine times and led to no significant reduction in virus infectivity. Therefore, H_2_O_2_ and MiST^®^ are not recommended for reducing airborne virus concentrations in occupied spaces.

The concentration of Isopropanol (from Pledge^®^) used in this study (16 ppm) is 25 times below the ACGIH TWA recommended guidelines (400 ppm, http://www.cdc.gov/niosh/idlh/67630.html). The concentration of the two other active ingredients in Pledge^®^ (n-Alkyl Dimethyl Benzyl Amonium Chlorides and n-Alkyl Dimethyl Ethylbenzyl Ammonium Chloride) was 2 ppm for both. The TWA guidelines for these quaternary ammonium salts are not available. The Eugenol concentration used in the current study was 16 ppm, however the TWA guidelines for this product has not been established. Since very low concentrations of the active ingredients can significantly reduce phage infectivity, Pledge^®^ and Eugenol can be tested in occupied spaces and could contribute to reducing the risk of viral transmission.

## Conclusion

The four phages used in this study displayed different resistance levels to RH and germicidal products once airborne. These results reiterate the idea that more than one viral agent is required to truly demonstrate the effectiveness of germicidal treatments. Pledge^®^ and Eugenol were the most effective products for reducing viral loads in aerosols although the active ingredients in Pledge^®^ responsible for these activities has not been elucidated. Large-scale studies in vacant and occupied spaces are currently being designed to investigate whether these products can be used to reduce airborne virus loads in indoor settings.
